# Injury-Associated PACAP Expression in Rat Sensory and Motor Neurons Is Induced by Endogenous BDNF

**DOI:** 10.1371/journal.pone.0100730

**Published:** 2014-06-26

**Authors:** Lina M. E. Pettersson, Nicole M. Geremia, Zhengxin Ying, Valerie M. K. Verge

**Affiliations:** CMSNRC & Department of Anatomy & Cell Biology, University of Saskatchewan, Saskatoon, SK, Canada; Hokkaido University, Japan

## Abstract

Peripheral nerve injury results in dramatic upregulation in pituitary adenylate cyclase activating polypeptide (PACAP) expression in adult rat dorsal root ganglia and spinal motor neurons mirroring that described for the neurotrophin brain derived neurotrophic factor (BDNF). Thus, we posited that injury-associated alterations in BDNF expression regulate the changes in PACAP expression observed in the injured neurons. The role of endogenous BDNF in induction and/or maintenance of PACAP mRNA expression in injured adult rat motor and sensory neurons was examined by intrathecally infusing or intraperitoneally injecting BDNF-specific antibodies or control IgGs immediately at the time of L4-L6 spinal nerve injury, or in a delayed fashion one week later for 3 days followed by analysis of impact on PACAP expression. PACAP mRNA in injured lumbar sensory and motor neurons was detected using in situ hybridization, allowing quantification of relative changes between experimental groups, with ATF-3 immunofluorescence serving to identify the injured subpopulation of motor neurons. Both the incidence and level of PACAP mRNA expression were dramatically reduced in injured sensory and motor neurons in response to immediate intrathecal anti-BDNF treatment. In contrast, neither intraperitoneal injections nor delayed intrathecal infusions of anti-BDNF had any discernible impact on PACAP expression. This impact on PACAP expression in response to BDNF immunoneutralization in DRG was confirmed using qRT-PCR or by using BDNF selective siRNAs to reduce neuronal BDNF expression. Collectively, our findings support that endogenous injury-associated BDNF expression is critically involved in induction, but not maintenance, of injury-associated PACAP expression in sensory and motor neurons.

## Introduction

The peptidergic phenotype in sensory and motor neurons is dramatically changed in response to nerve injury and is implicated in the neurons’ capability to survive and regenerate. However, the signals inducing this phenotypic switch, to a regenerative state, are relatively unknown. We and others have previously shown that one of these peptidergic changes is an injury-induced expression of the neuropeptide pituitary adenylate cyclase activating polypeptide (PACAP) in both sensory and motor neurons. These changes in PACAP expression parallel changes that we have observed in brain derived neurotrophic factor (BDNF) in the injured neurons over the same time frame. The aim of this study was to examine our hypothesis that injury-induced changes in BDNF expression regulate induction of injury-associated PACAP expression in sensory and motor neurons.

In sensory and motor neurons PACAP expression is markedly upregulated in response to various types of nerve damage (see below) where it serves roles in modulation of nociception [1]–[6], regeneration and survival of injured neurons [7]–[11]. DRG neurons are immunoreactive for PACAP and express PACAP mRNA, whereas in the spinal cord, PACAP immunoreactivity has been found in nerve fibers in the superficial laminae of the dorsal horn, dorso-laterally to the central canal [12]–[17], and also in fibers and neurons in the intermediolateral column (IML) [14], [18]. Expression of PACAP mRNA has been observed in cell somas primarily in the superficial layers of the dorsal horn, but also in some neurons around the central canal, in motor neurons in the ventral horn [19], and in neurons in the IML [18]. Interestingly, expression of the PACAP preferring receptor, PAC_1_, is detectable in spinal cord dorsal and ventral horn neurons but not in DRG neurons, suggesting a paracrine role for primary sensory neurons [16]. Under homeostatic conditions, roughly a fifth of the rat DRG neurons (primarily nerve cell bodies of smaller diameter) express mRNA or show immunoreactivity for PACAP, whereas very few spinal cord ventral horn neurons show PACAP expression [12]–[16], [20]. However, this expression is highly plastic and changes in response to nerve lesion or inflammation. After sciatic or spinal nerve transection PACAP expression is induced in spinal cord motor neurons as well as in DRG neurons [13], [19]. In the DRG, a phenotypic switch is observed whereby expression is induced primarily in medium-large diameter neurons, while expression in the small size neurons declines after sciatic or spinal nerve transection [13], [15], [16], [21]. This contrasts with what is observed in response to a compression injury where an upregulation in PACAP expression is observed across all size ranges of DRG neurons [22].

Neurotrophins are potent modulators of neuropeptide expression in sensory neurons with three members having been studied in this capacity – nerve growth factor (NGF), neurotrophin 3 (NT-3) and BDNF [23], [24]. We have previously shown that both NGF and NT-3 are able to modulate PACAP expression in DRG neurons [21], [25]. NT-3 downregulates PACAP mRNA expression in intact DRG neurons and mitigates the increased expression in large neurons after proximal nerve transection, whereas NGF promotes an upregulation of PACAP mRNA expression in intact as well as transected or inflamed small size DRG neurons [21], [25]. While the sources of NGF and NT-3 available to sensory neurons are primarily target-derived, BDNF differs in that it is also expressed in the DRG in subpopulations of sensory neurons consistent with those that express PACAP both before, and following injury [21], [26], [27]. Normally BDNF is expressed in ∼30% of primarily small-size DRG neurons, but is upregulated in response to nerve transection in a pattern that mirrors that described for PACAP [26], [27]. In the spinal cord, BDNF mRNA is expressed in ∼73% of the lumbar motor neurons, and there is a corresponding protein expression in these neurons [28]. The expression is upregulated in response to femoral or sciatic nerve transection and ventral root avulsion [29]–[31] and has been shown to modulate repair programs in the central nervous system as well as in motor neurons [32]–[34]. The precise role(s) of injury-induced alterations in neuronal BDNF expression for the peripheral nervous system remains largely elusive. However, we have recently shown that endogenous BDNF regulates induction of regeneration-associated gene expression (growth-associated protein-43 and Tα1 tubulin) and the intrinsic ability of injured sensory neurons to extend neurites in response to spinal nerve injury [35]. Whether it serves a similar role in regulating injury-associated changes in peptide expression in sensory and motor neurons is unknown. The present study examines this and identifies BDNF as an endogenous regulator of induction but not maintenance of PACAP mRNA expression in injured sensory and motor neurons after sciatic spinal nerve transection.

## Materials and Methods

### 1. Experimental animals, models and tissue processing

#### Ethics statement

All animal procedures were conducted under the approval of the Animal Research Ethics Board at the University of Saskatchewan (protocol number: 19920164) and in accordance with the Canadian Council on Animal Care. All efforts were made to minimize suffering and animals were given buprenorphine (Temgesic; 0.05–0.1 mg/kg) subcutaneously pre- and postoperatively.

#### 1.1. Experimental setup and anti-BDNF treatment

Male Wistar rats (∼250–300 g) were anaesthetized with sodium pentobarbital (Somnitol, 65 mg/kg, MTC Pharm, Canada), and all animals underwent unilateral sciatic spinal nerve transection, whereby the right sciatic nerve was transected, at its origin from the L4, L5 and L6 spinal nerves, and a 5 mm segment resected to prevent regeneration. Animals were divided into two groups –those receiving continous 3 day intrathecal infusion or those receiving intraperitoneal injections (i.p.) of anti-BDNF or control IgG. In a previous study we confirmed that the antibody employed is highly selective for BDNF and does not recognize other neurotrophins, nor cytochrome C a protein of similar molecular weight and isoelectric point [35], in agreement with others [36]–[38].

*Intrathecal infusion group:* Animals were given continuous intrathecal infusions of sheep anti-BDNF (Chemicon International, Temecula, CA, USA; diluted in sterile distilled water) or control IgG (Sigma-Aldrich, St. Louis, MO, USA) either immediately (anti-BDNF, n = 6; control IgG, n = 6) or 7 days after injury (referred to as “delayed”; anti-BDNF, n = 7; control IgG, n = 7) at 1.5 µg/µl/hour for 3 days (dose based on Zhou et al., 2000 [39]), via mini-osmotic pumps (Alzet model 2001, Durect Corporation, Cupertino, CA, USA). The pump was inserted into the dorsal lumbar subcutaneous space and attached to a silicone tubing (0.3 mm outer diameter) that was passed through the dura and arachnoid at the lumbo-sacral junction and ran cranially along the spinal cord for approximately 1.5 cm, delivering the antibody solution at the level of the L4-L6 DRGs.

*Intraperitoneal injection group:* Animals were given systemic injections of 0.75 ml anti-BDNF solution (n = 3; 1 mg/ml in sterile distilled water) or sheep control IgG (n = 3), (dose based on Zhang et al., 2000 [38]), 1 hour before, and 24 and 48 hours after the injury.

Animals treated with control IgG served as controls for the anti-BDNF treated animals, while injury alone animals i.e. injured animals receiving no infusions (n = 11) were used to assess the impact of infusing/injecting IgGs into the animal. Three days after introduction of the pump or injections, animals were anaesthetised and perfused (see below).

#### 1.2. siRNA treatment

*In vivo* reduction of neuronal BDNF expression by small interfering RNAs (siRNA), was employed as an additional method by which to interfere with endogenous BDNF as in [35] and adapted from Baker-Herman et al. (2004) [40]. siRNA directed against rat BDNF mRNA (BDNF siRNA; accession number – M-012513), a control non-targeting scrambled sequence (non-targeting control siRNA; Pool #D-001206-13-20; 5′-AUGUAUUGGCCUGUAUUAG-3′; 5′-UAGCGACUAAACACACAUCAA-3′) and a fluorescently labeled control scrambled siRNA (siGLO; – RISC–free siRNA #D-001206-13-20; proprietary sequences) were obtained from Dharmacon Inc, Lafayette, CO, USA. BDNF siRNA was comprised of 4 pooled 21-nucleotide duplexes with symmetrical 3′ overhangs and a 5 phosphate on the antisense strand (SMARTpool - #M-080046–00): 1) 5′-PUCAUCCAGCAGCUCUUCGAUU, 2) 5′-PUUAAUGGUCAGUGUAC. AUAUU, 3) 5′-PAAUACUGUCACACGCUCUU and 4) 5′-PACAUACGAUUGGG. UAGUUCUU. All siRNAs were diluted with siRNA Universal Buffer (Dharmacon Inc.) to a concentration of 100 µM. Aliquots of siRNA stock were stored at −80°C until use.

To deliver the siRNAs, a laminectomy was performed at the lumbar sacral junction and a sterile indwelling catheter inserted into the subarachnoid space such that the tip of the catheter lay along the midline at the level of the L5 DRG. siRNA (20 µg - 15 µl siRNA from the 100 µM stock) was combined with 2 µl oligofectamine (Invitrogen) and incubated for 15 minutes at room temperature prior to intrathecal injection over a 5 minute span, followed by 3 µl of sterile PBS to flush the catheter. This first injection was performed to help deplete existing stores of BDNF protein that could be released upon injury. Three days later the same animals were subjected to a second injection of BDNF siRNA (n = 2) or control non-targeting siRNA (n = 2) which was administered via the indwelling catheter immediately followed by unilateral transection of the L4, 5, 6 spinal nerves. Fluorescently labeled control scrambled siRNA – siGLO (n = 2) were also delivered intrathecally in an identical manner to BDNF siRNA and non-targeting control siRNA to visualize and ascertain the cellular uptake within the DRG. An additional 4 control animals received unilateral transection of the L4, 5, 6 spinal nerves alone. Three days later animals were perfused and DRGs (L5) removed and processed for in situ hybridization to detect PACAP mRNA and assess the impact of BDNF silencing on PACAP expression.

The efficacy of BDNF siRNA treatment in reducing BDNF mRNA and protein expression in DRG neurons was assessed, using BDNF in situ hybridization and immunohistochemistry respectively.

#### 1.3. Tissue processing

On the day of dissection, animals were deeply anaesthetized and perfused via the aorta with cold phosphate buffered saline (PBS, 0.1 M, pH 7.4), followed by 4% paraformaldehyde (PF) in phosphate buffer (PB, 0.1 M, pH 7.4). Dissected tissues (lumbar spinal cord, and L4, 5 DRGs) were postfixed (1–1.5 hours) and cryoprotected in 20% sucrose. Control and experimental tissues were embedded in the same cryomolds, covered in OCT and frozen in cooled isopentane prior to storing at −80°C until processing.

Sections were analysed and photographed with an Olympus BX-60 microscope connected to an Olympus DP-50 digital camera. Brightness and contrast were adjusted with the software Adobe Photoshop 5.0.

### 2. Immunohistochemistry

#### 21. Detection of injured neurons

Injured neurons in spinal cord sections were identified using an antibody against activating transcription factor 3 (ATF-3), a specific marker induced in injured DRG and motor neurons [41]. The tissues were sectioned serially at 6 µm in a cryostat, with one section processed for ATF-3 immunohistochemistry while the adjacent section was processed for in situ hybridization for detection of PACAP mRNA, see below. For immunohistochemistry slides were air-dried, washed in phosphate buffered saline (PBS, 3×10 minutes) and blocked with 2% horse serum in 0.1% triton-X in PBS for 1 hour at room temperature. After blocking, tissues were incubated with primary antibody, rabbit anti-ATF-3 (sc-188, diluted 1∶300 in the blocking solution; Santa Cruz Biotech Inc, Santa Cruz, CA) overnight in air sealed, humidified containers at 4°C. The following day, slides were washed in PBS (3×10 minutes) and incubated with secondary antibody, donkey anti-rabbit Fab2 Cy3 conjugated (711-166-152, diluted 1∶300 in PBS; Jackson ImmunoResearch, Westgrove, PA) for 1 hour in the dark at room temperature. Finally, slides were washed in PBS (3×10 minutes) and coverslipped with glycerol/PBS (1∶1). To establish the specificity of the immunostaining, additional slides were incubated with the omission of primary antibody and processed as above. The specificity of the ATF-3 antiserum has previously been established by Western blot where it bound to its cognate peptide and presented a band at the proper molecular weight [42], as well as with immunohistochemistry on DRG tissue after absorption with its blocking peptide in excess after which staining was abolished [43].

#### 2.2. Infused antibody distribution

DRG and spinal cord sections were, rinsed in PBS, and incubated with biotinylated-anti-sheep IgG (1∶150; Vector, Burlington, ON, Canada) for 1 hour at room temperature. Sheep IgG distribution was visualized in target tissues using a standard avidin/biotin/peroxidase procedure as per manufacturer’s instruction (Vectastain ABC Elite kit; Vector Laboratories, Canada).

#### 2.3. BDNF immunohistochemistry

BDNF immunohistochemistry was performed essentially as decribed in Geremia et al., [35] employing a rabbit anti-BDNF (1∶500; kind gift of Dr. C. Wetmore [44]).

### 3. *In situ* hybridization

Oligodeoxyribonucleotide (OligoDNA) probes complementary to and selective for PACAP mRNA -nucleotides 700–747 [45], rat BDNF (1) complementary to bases 213**–**260 [46]; GenBank accession number **–** M61175, rat BDNF (2) complementary bases 626**–**673 [47]; GenBank accession number **–** X16713 were synthesised and purified (University of Calgary DNA services, Alberta, Canada). All cDNA regions used were checked against the Genbank database (NIH, at the Internet site www.ncbi.nlm.nih.gov); no greater than 60% homology were found to sequences other than the cognate transcript. Labeling of probe (at a concentration of 3.1 ng probe/µl), with ^35^S–dATP (Perkin Elmer, Waltham, MA, USA) and terminal transferase enzyme (Amersham, Canada) was performed in a terminal transferase buffer, containing sodium cacaodylate 500 mM, CoCl_2_ (pH 7.2) 10 mM, mercaptoethanol 1 mM, for 1.5–2 hours at 37°C. The reaction was stalled by adding 500 µl, 0.1 M Tris HCl (pH 8.0), after which probe was purified through a NENSORB-20 column (New England Nuclear, Boston, MA, USA), and dithiothreitol added to a final concentration of 10 mM. The activity was measured to approximately 0.27–0.28×10^9^ cpm/ml, and the labeled probe was stored at 4°C.

All steps prior to hybridization were performed under RNase free conditions, and all dilutions were performed in autoclaved double distilled water. The slides were air-dried and postfixed in 4% PF (20 minutes), washed in PBS (3×5 minutes), treated with proteinase K at 37°C (20 µg/ml; 7–8 minutes), rinsed in PBS (5 minutes), fixed in 4% PF (5 minutes), rinsed in PBS (2×5 minutes), rinsed in diethyl pyrocarbonate (0.1%) -H_2_O (5 minutes), and dehydrated in increasing ethanol concentrations (70%, 90%, 100%; approximately 1 minute in each). Sections were hybridized with radiolabeled probe at a concentration of 10^7^ cpm/ml in a hybridization solution consisting of 50% formamide, 4×saline sodium citrate (SSC; 1×SSC = 0.15 M NaCl, 0.015 M sodium citrate), 1×Denhardts solution (0.02% bovine serum albumin (BSA), 0.02% Ficoll and 0.02% polyvinylpyrrolidone), 10% dextran sulphate, 0.5 mg/ml salmon sperm DNA, 1% sarcosyl and 0.2 M DTT. Hybridization, with approximately 100 µl hybridization solution/slide, was conducted over night at 43°C in air sealed, humidified chambers to prevent evaporation. Following hybridization, the slides were washed in 1×SSC (4×15 minutes, 55°C, and an additional 30 minutes, room temperature), dipped twice in distilled water, dehydrated in ascending ethanols and air dried. Slides were dipped in Kodak NTB2 photoemulsion (diluted 1∶1 in distilled water), to generate autoradiograms. After 4.5–10 weeks exposure the slides were developed in Kodak D19 (3–5 minutes), rinsed in water, fixed in Kodak rapid fix (5 minutes) and rinsed in water (20 minutes). For darkfield viewing and photography slides were left unstained, whereas slides for brightfield examination were counterstained with 0.5 % toluidine blue (in an acetate buffer; pH 4–4.5), and mounted with Permount (Fisher, Canada). The specificity of hybridization signal for the individual probes was determined by hybridization of adjacent 6 micron sections with labeled probe with the addition of either 1000-fold excess corresponding unlabeled probe which abolished the signal, or 1000-fold excess of a dissimilar unlabeled probe of the same length and similar G-C content which left the signal unchanged from that observed with labelled probe alone.

### 4. Quantification and image analysis

#### STEP I

To evaluate the impact of immunoneutralizing or reducing endogenous BDNF expression on PACAP mRNA expression in axotomised DRG neurons, slides from all animals were first analysed qualitatively to ensure that consistent alterations in expression between experimental groups were observed. All slides examined had both experimental and control tissues mounted on the same slide, thus avoiding bias induced by slide to slide variability in hybridization efficiency and allowing for an accurate determination of relative changes between experimental groups. Qualitative analysis was performed where the relative changes in hybridization signal between experimental groups mounted on the same slide were noted. Representative slides were selected for quantitative analysis based on the criterion that all DRG sections on that slide contained similar numbers of neurons. Photomontages of DRGs for all experimental groups mounted on the same slide were prepared and individual neurons with a visible nucleus were identified and numbered. All neurons within individual DRGs on the same slide were assessed (using a 63X objective) as being labeled, i.e. having levels of hybridization signal above background or not, thus yielding information on incidence of expression. In this manner approximately 820 neurons per experimental group were quantified for immediate infusion of anti-BDNF and control IgG groups and approximately 495 neurons per experimental group were analysed for i.p. injections.

#### STEP II

Only immediate intrathecal infusion of anti-BDNF (but not i.p. injections) had a discernible impact on the incidence and the degree of expression per individual neuron, the latter assessed qualitatively. Therefore only intrathecally infused animals were subjected to additional computer-assisted image analysis. Further, assessment of impact of treatment on PACAP expression in STEP I revealed neither discernible changes in the level of PACAP hybridization signal between injury alone and control IgG-infused injured tissue, nor any effect on incidence of expression between these two control groups. Thus, only control IgG- and BDNF IgG-infused groups were examined quantitatively using computer-assisted image analysis. An additional two animals from each of the immediately infused experimental groups were selected for this form of analysis bringing the total number of animals quantitatively assessed to 4 for each experimental group. In addition, quantification of PACAP mRNA expression in tissues from animals receiving delayed intrathecal infusions was also performed (n = 3 animals/treatment group).

The proportion of labeled neurons, as well as the level of expression in each neuron and the distribution of labeling in relation to neuronal size, i.e. in small vs medium-large diameter neurons were determined. For each DRG analysed montages were made, neurons with a visible nucleus identified and each individual neuron was analysed using computer assisted image analysis. This form of analysis was performed for animals receiving immediate intrathecal infusions - 2 rats infused with anti-BDNF and 2 rats infused with control IgG with ∼225 neuronal profiles/L5 DRG section being examined for a total of ∼450 neurons per experimental group. It was also performed on animals receiving delayed intrathecal antibody infusions - 3 rats infused with anti-BDNF and 3 rats infused with control IgG with ∼300 neuronal profiles/L5 DRG section were examined. In short, cross sectional areas and the percentage of the cytoplasmic area covered by silver grains were measured for all the identified neurons in all the ganglia, using the public domain NIH Image program (written by Wayne Rasband at the US National Institute of Health, (zippy.nimh.nih.gov). In DRG, neurons were readily distinguished from glial cells (i.e. perineuronal satellite glial and Schwann cells) based on histological criteria in the toluidine blue stained sections with neurons exhibiting lightly stained nuclei and a stained cell soma, in contrast to the glial cells which appear as darkly stained, more oblong nuclei and are significantly smaller in size [48].

Analyses of DRG neurons were further subdivided into small (diameter <35 µm), and medium-large (diameter ≥35 µm) [49]. A total of ∼900–1300 DRG neuronal profiles were analysed per treatment group.

For spinal cord ventral horn neurons, a similar approach was used. While spinal nerve lesion injures virtually all the DRG neurons, the injured motor neurons need to be identified in an adjacent section by the presence of nuclear ATF-3 immunoreactivity, which is an injury marker. Only motor neurons identified as injured were quantified. Montages were prepared from adjacent sections (hybridized for detection of PACAP mRNA and ATF-3 immunohistochemistry), and all neurons showing nuclear ATF-3 immunoreactivity were analysed to determine the density of PACAP mRNA hybridization signal over the injured motor neuron.

Neurons were considered labeled for PACAP mRNA if they contained more than 4 or 6 times background labeling of silver grains for ventral horn and DRG neurons, respectively. This threshold corresponds to a level of silver grains that must be present to confidently identify the neuron as positively labeled when examined under 63 X oil immersion as in STEP I of the analysis. Background levels of hybridization signal were determined by averaging the silver grain density over 5–10 defined areas of the neuropil devoid of positively labeled cell bodies for each section being analysed.

Differences in the proportions of PACAP mRNA expressing neurons between the control IgG treated and anti-BDNF treated DRGs, were analysed using the chi-square test, with p≤0.05 being considered significant, whereas differential induction of PACAP mRNA in injured motor neurons were analysed using the Mann Whitney U test and p≤0.05 was considered significant.

#### 4.1. Real-Time Quantitative Reverse Transcription PCR (qRT-PCR) analysis

Total RNA was isolated from the L4 and L5 DRGs using the RNeasy Kit (Qiagen, Toronto, ON, Canada). The quality of RNA was analyzed by electrophoresis using the Agilent 2100 bioanalyzer (Agilent Technologies, CA). The RNA was converted to cDNA using the QuantiTect Rev. Transcription Kit (Qiagen, Toronto, ON, Canada). The PACAP cDNA was amplified by primers 5**′**- GCCTACGCCCTTTACTACCC -3**′** and 5**′**- GAAGATGCCGTCCGAGTG -3**′** with a qRT-PCR machine (Stratagene, CA). The levels of housekeeping gene GAPDH (amplified by primers 5**′**- GGTGCTGAGTATGTCGTGGAGTC-3**′** and 5**′**- GTGGATGCAGGGATGATGTTCT-3**′** ) were used as internal control to verify the qRT-PCR reaction. A non-template control was included in each set of reactions.

Statistical analyses were performed with Prism (GraphPad Software, Inc.). Differences between experimental group means were assessed by one-way analysis of variance (ANOVA) with post hoc Tukey's analysis. Differences were considered significant at p≤0.05.

## Results

### 1. Penetration of intrathecally infused anti-BDNF and control IgGs into surrounding nervous tissues

**Figure 1:**
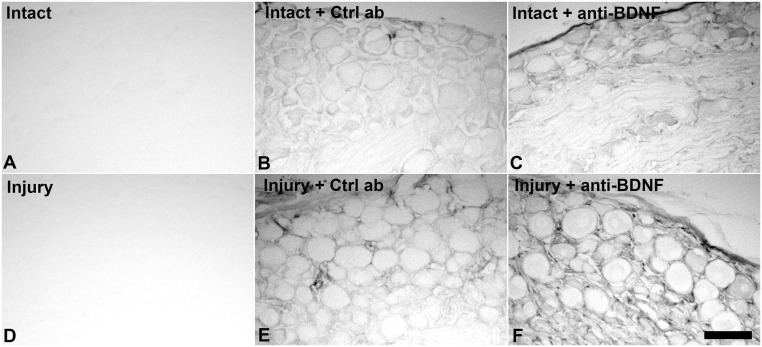
Intrathecally infused anti-BDNF and control IgGs effectively penetrate DRG tissue. Brightfield photomicrographs of L5 DRG sections with or without antibody infusions, depicting antibody penetration into intact DRG or 3 days following sciatic spinal nerve injury. Both control sheep IgGs and sheep anti-BDNF antibodies readily penetrate the tissue after intrathecal delivery showing immunoreactivity both in the parenchyma and the surrounding connective tissue in intact (B, C) and injured (E, F) animals. Note, no staining for sheep IgGs is found in DRGs from animals not receiving antibody infusions (A, D). Scale bar 100 µm.

To determine whether intrathecally infused antibodies effectively penetrate the surrounding DRGs and corresponding regions of the spinal cord, these tissues were processed immunohistochemically to detect sheep IgGs. DRG sections from antibody infused (control IgG or anti-BDNF) animals revealed immunoreactivity in the parenchyma as well as the surrounding connective tissue in lumbar DRG contralateral and ipsilateral to axotomy (Fig. 1B, C, E, F), whereas no staining was found in tissues from animals without infusions (Fig. 1A, D). The staining intensity was strongest in the injured DRGs, most likely due to an increased permeability of the blood/nerve barrier caused by the nerve lesion [50]. Thus, intrathecally delivered antibody effectively penetrated into the ganglia. However, it was not readily detected in the spinal cord dorsal or ventral horns at the lumbar enlargement, the level where the afferents/efferents from the injured neurons enter/exit the spinal cord (data not shown).

### 2. Role of endogenous BDNF in induction of PACAP mRNA expression in sensory and motor neurons following spinal nerve transection

**Figure 2:**
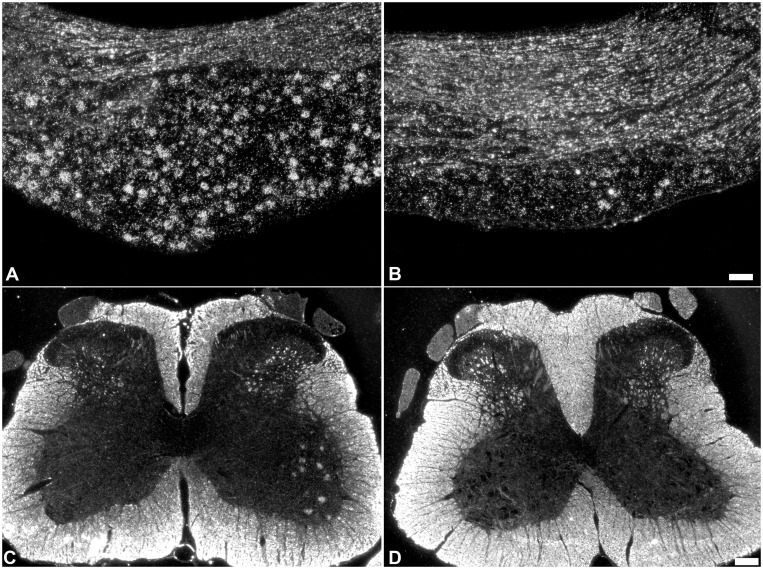
PACAP mRNA expression is reduced in injured sensory and motor neurons after immediate anti-BDNF infusion. Darkfield photomicrographs of PACAP mRNA hybridization signal over sensory neurons in L5 DRG sections (A, B) and spinal cord motor neurons (C, D) in response to 3 days sciatic spinal nerve transection in conjunction with immediate 3 days infusion of intrathecal control IgG (A, C) or anti-BDNF (B, D) infusions. The increase in PACAP mRNA expression observed after nerve transection is markedly reduced by anti-BDNF infusion. Both the incidence of neurons expressing detectable PACAP mRNA and the level of expression in individual neurons are decreased. Scale bar (A, B) 100 µm, (C, D) 200 µm.

**Figure 3:**
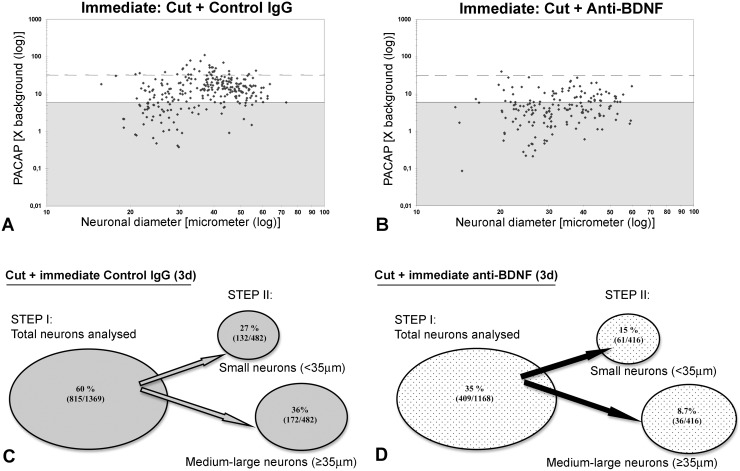
Incidence, level and distribution of PACAP mRNA expression in DRG neurons after immediate antibody infusion. Representative scatterplots depicting relative changes in PACAP mRNA hybridization signal over individual neurons in relation to neuronal size after sciatic spinal nerve transection and immediate intrathecal control IgG (A), or anti-BDNF (B) infusion. Each dot represents signal intensity over an individual neuron as a function of neuronal size. Solid lines divide the plots into unlabeled (shaded), and labeled (≥6 X background labeling) populations, and dashed lines separate moderately from highly labeled (≥32 X background) neuronal populations. (C, D) STEP I -incidence of PACAP expression was determined for all neurons analysed for the Control IgG infused group (C; n = 4 animals or 1369 neurons) or anti-BDNF infused group (D; n = 4 animals or 1168 neurons). STEP II –impact of treatment on the incidence of PACAP expression in small (<35 µm) versus medium-large (>35 µm) DRG neurons was determined for immediate intrathecal infusions of either Control IgG (C; n = 2 or 482 neurons) or anti-BDNF (D; n = 2 or 416 neurons) for the animals that had undergone computer-assisted image analysis. Note: the injury-induced increase in PACAP mRNA expression is significantly prevented by immediate intrathecal anti-BDNF infusion (p<0.001, chi-square test). A reduction is observed both in the number of small and medium-large diameter DRG neurons expressing detectable PACAP mRNA, as well as at the level of hybridization signal/neuron.

We wanted to determine whether endogenous BDNF is involved in the regulation of PACAP mRNA expression after nerve injury. Thus, the impact of immediate intrathecal infusion of sheep anti-BDNF or sheep IgG, for 3 days at the time of sciatic spinal nerve transection, on PACAP mRNA expression was investigated. The 3 day infusion/injury period was chosen since BDNF expression is elevated in ∼80% of the injured sensory neurons at this timepoint [26]. Examination of the slides revealed that the distribution of PACAP mRNA expression in intact sensory neurons (primarily small size neurons) contralateral to the injury is in agreement with previous studies and represents ∼22% of sensory neurons [15], [16], [21], [22], [25]. Sciatic spinal nerve injury resulted in elevated expression of PACAP mRNA in both 3 day sciatic spinal nerve injured DRG neurons (data not shown) and 3 day injured with 3 day infusion of control IgG in conjunction with the injury (Fig. 2A) relative to contralateral uninjured DRG. Three day spinal nerve transection resulted in ∼60% of the L5 DRG neurons expressing detectable PACAP mRNA, also in agreement with previous findings [16]. A similar increased incidence and level of PACAP mRNA expression was found in L5 DRG neurons ipsilateral to lesion in the control IgG infused group (Fig. 3C). Since no discernible differences were observed between the control IgG and injury alone groups, quantitative analyses were performed between control IgG and anti-BDNF treated animals.

Immunoneutralization of endogenous BDNF by immediate intrathecal infusion of anti-BDNF had a dramatic impact on the induction of PACAP expression in 3 day injured sensory neurons, resulting in decreased incidence and level of PACAP mRNA expression (Fig. 2B and Fig. 3B, D) with only one third of the injured DRG neurons expressing detectable PACAP mRNA. The reduced incidence of expression was observed across all size ranges of neurons after immunoneutralization (Fig. 3). In addition, there was a marked decrease in the numbers of neurons expressing PACAP mRNA at high levels (>32x background) after anti-BDNF infusion, with only the rare neuron appearing to do so (Fig. 3B). Thus, a significant difference in both the number of neurons, as well as the level of PACAP mRNA expression/neuron, was established when anti-BDNF treated DRGs and DRGs treated with control IgG were compared after injury.

Uninjured lumbar motor neurons show negligible levels of PACAP mRNA expression. L4-6 spinal nerve injury however, induced, PACAP mRNA expression in lumbar motor neurons, in agreement with what we have previously shown [19]. No discernible impact on this response was observed when control IgG was infused in conjunction with the injury. Immunoneutralization of endogenous BDNF reduced the level and incidence of PACAP expression in axotomized motor neurons from ∼55% of the injured motor neurons expressing PACAP mRNA in control IgG infused animals, to ∼35% of the neurons after anti-BDNF infusion (Fig. 2C, D). When the level of PACAP mRNA expression per individual axotomized motor neuron was examined, levels ranged from 6.0–37 times the background expression (median 11) in control IgG treated animals, whereas the levels were much lower after anti-BDNF treatment, ranging from 4–10 times background expression (median 8).

### 3. Role of endogenous BDNF in maintaining PACAP mRNA expression in injured sensory and motor neurons after spinal nerve transection

**Figure 4:**
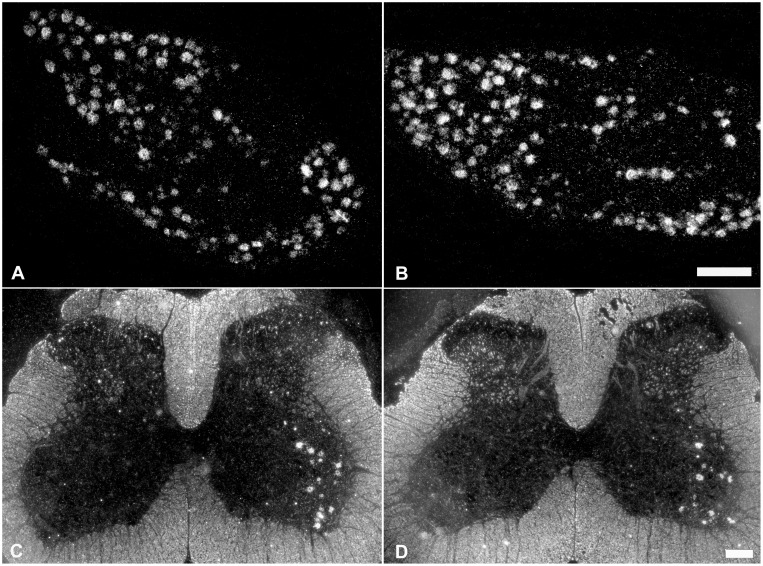
Neuronal injury-induced upregulation of PACAP mRNA expression is not discernibly impacted by delayed anti-BDNF infusion. Darkfield photomicrographs depicting PACAP mRNA expression in DRG neurons (A, B) and spinal cord motor neurons (C, D) after 10 day sciatic spinal nerve transection in conjunction with delayed intrathecal control IgG (A, C) or anti-BDNF (B, D) infusions for 3 days, 7 days after injury. Note, delayed anti-BDNF infusion does not visibly affect PACAP mRNA signal intensity over DRG or spinal cord motor neurons. Scale bar 200 µm.

**Figure 5:**
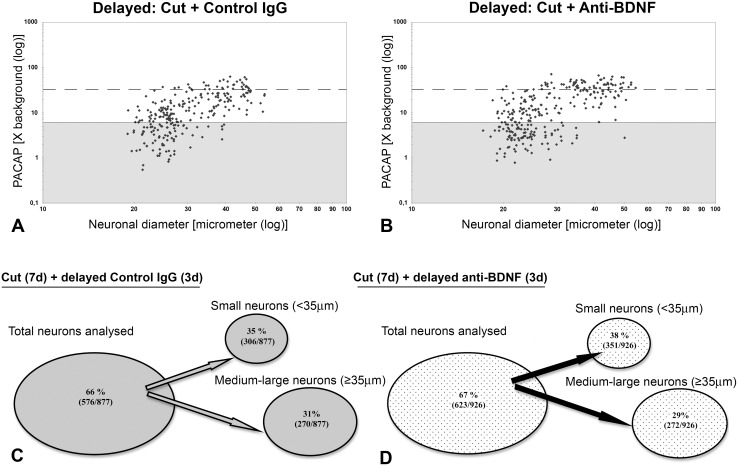
Incidence, level and distribution of PACAP mRNA expression in DRG neurons after delayed antibody infusion. Representative scatterplots depicting relative changes in PACAP mRNA hybridization signal over individual neurons in relation to neuronal size after delayed control IgG (A) or anti-BDNF (B) infusions. Each dot represents hybridization signal intensity over a quantified neuron. Solid lines divide the plots into unlabeled (shaded) and labeled (≥6X background labeling) populations, and dashed lines separate moderately from highly labeled populations (≥32 X background). Numbers and percentages of DRG neurons expressing detectable PACAP mRNA after delayed intrathecal control (C) or anti-BDNF (D) antibody treatment for 3 days following 7 days of injury (n = 6, 3 animals analysed per treatment group). Note, delayed intrathecal anti-BDNF infusion did not have any significant effect on the injury-induced increase in PACAP mRNA expression after axotomy.

Delayed immunoneutralization of endogenous BDNF by intrathecal infusion of BDNF antibodies for 3 days beginning at 1 week after injury (10 day injury period) did not have any discernible effect on injury-induced PACAP mRNA expression in DRG or spinal cord motor neurons (Fig. 4 and Fig. 5). PACAP mRNA was expressed in 66% and 67% of the 10 day injured DRG neurons after the delayed intrathecal control IgG or anti-BDNF infusions, respectively. Furthermore, the proportion of small and medium-large size DRG neurons expressing PACAP mRNA was very similar after delayed control IgG and anti-BDNF infusions (Fig. 5). Data from the infused animals were also compared with findings from animals receiving injury alone, and no obvious differences were detected. The injury-induced expression of PACAP mRNA in spinal cord motor neurons was also comparable after delayed infusion of control IgG and anti-BDNF when 69% and 67% of the injured (ATF-3 positive) neurons expressed PACAP mRNA, respectively (Fig. 4C, D). Consistent with sensory neurons, the proportion of injured motor neurons expressing PACAP mRNA after injury alone (63%) and control IgG infusion (69%) was not significantly different.

### 4. Confirmation of BDNF regulation of injury-induced PACAP expression using alternate approaches

**Figure 6:**
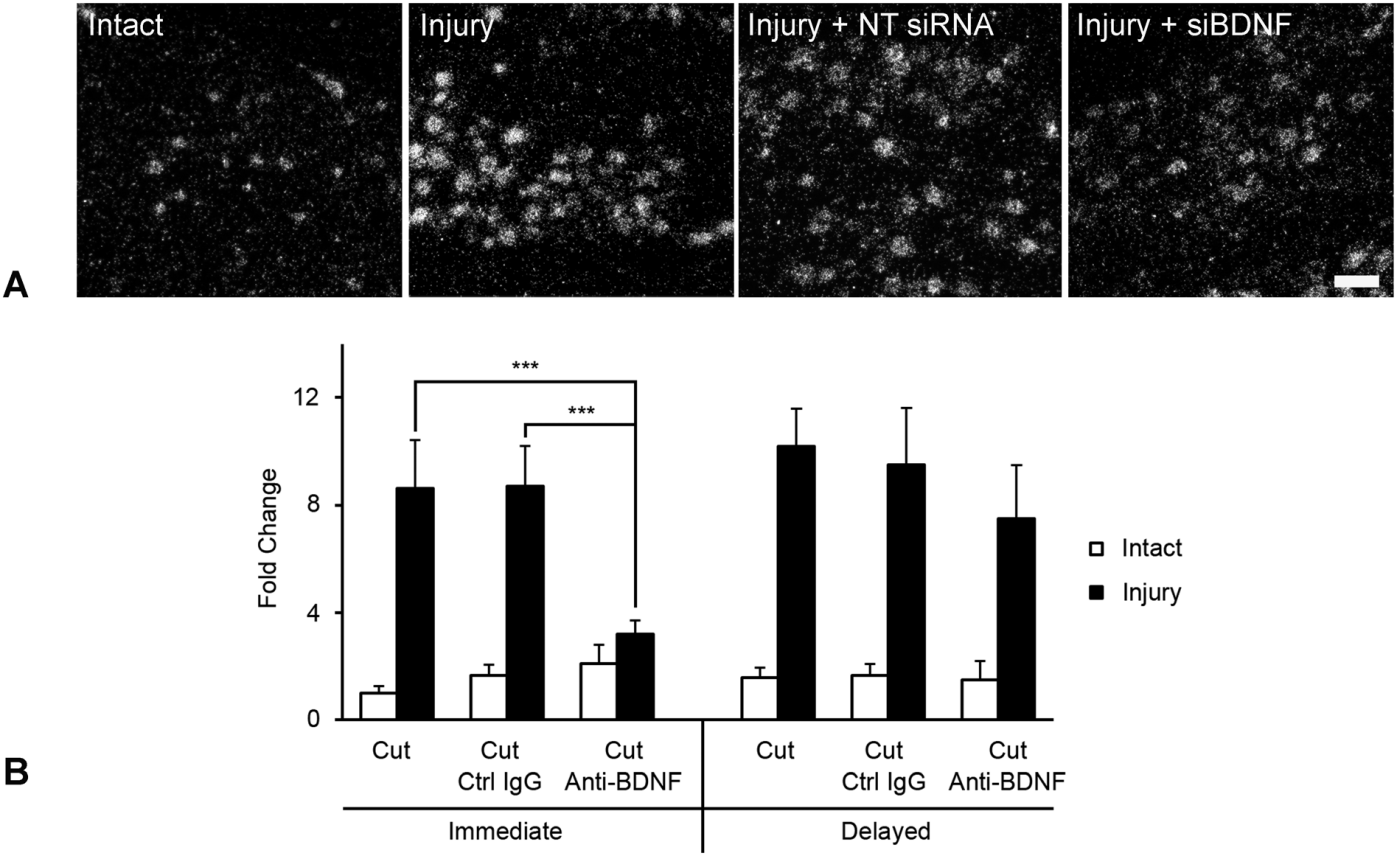
Alternate approaches supporting BDNF regulation of injury-associated PACAP expression. (A) Impact of BDNF siRNA infusion on PACAP mRNA expression in injured DRG neurons. Darkfield photomicrographs of L5 DRG sections processed for in situ hybridization to detect PACAP mRNA in intact sensory neurons (Intact), in association with 3d sciatic spinal nerve injury (Injury), 3d injury + infusion of either non-targeting control siRNA (Injury + NT siRNA) or siRNA targeting BDNF mRNA (Injury + siBDNF) as indicated. Note, there is no detectable difference in injury-induced PACAP expression after infusion of non-targeting control siRNA as compared to the Injury alone group, whereas animals treated with siBDNF, display decreased neuronal PACAP expression. Scale bar 100 µm. (B) qRT-PCR analysis of mRNA samples extracted from L4,5 DRG having undergone unilateral sciatic spinal nerve injury with or without either immediate or one week delayed 3 day intrathecal infusion of anti-BDNF or control IgG (Ctrl IgG) as indicated (performed in triplicate). Note that immediate anti-BDNF treatment significantly impairs the induction of injury-associated PACAP expression, while control IgG infusion has no effect. Delayed anti-BDNF does not significantly impact maintenance of PACAP expression in injured sensory neurons (***p<0.001).

qRT-PCR served as an alternate way to quantitatively assess fold change in mRNA expression between experimental and control conditions, but does so without information about impact on select neuronal subpopulations. We performed these additional experiments in triplicate using immediate or delayed intrathecal anti-BDNF delivery to immunoneutralize endogenous BDNF actions and assess the impact of this treatment on PACAP mRNA expression in injured DRG. Figure 6B reveals that anti-BDNF had an identical impact on PACAP mRNA expression in injured DRG as was determined using in situ hybridization. Examination of growth-associated protein-43 mRNA regulation using qRT-PCR analysis served as a positive control that the anti-BDNF treatment had worked (data not shown), It revealed a similar regulation to that observed for PACAP and confirmed our previous findings [35].

Finally, *in vivo* reduction of neuronal BDNF expression using BDNF selective siRNAs was adapted from Baker-Herman et al. (2004) [40] and performed as in [35]. Intrathecal delivery of fluorescently tagged siRNA at the level of the L5 ganglia resulted in an accumulation of fluorescence in L5 DRG neurons both ipsi- and contralateral to the injury, but was not detected in perineuronal nor other glial cells. The BDNF-selective siRNA was effective at reducing levels of detectable BDNF mRNA and protein expression in L5 sensory neurons in a manner equivalent to that previously shown, while control siRNAs had no discernible effect [35]. Thus, BDNF siRNA or non-targeting control siRNA were injected prior to and at the time of spinal nerve injury with impact on PACAP mRNA expression being examined 3 days later. The PACAP mRNA upregulation induced by nerve injury was not discernibly altered by infusion of non-targeting control siRNA, but was noticeably reduced after BDNF siRNA delivery (Fig. 6A), albeit not as robustly as following antibody infusion.

### 5. Impact of systemically delivered anti-BDNF or control IgG on injury-associated neuronal PACAP mRNA expression

In contrast to anti-BDNF that was intrathecally delivered immediately following injury, i.p. injections of anti-BDNF also delivered immediately following injury did not discernibly alter either the incidence nor the level of PACAP hybridization signal observed over injured sensory neurons when examined 3 days later. After i.p. injection of anti-BDNF, 67% of injured DRG neurons expressed detectable PACAP mRNA hybridization signal as compared the 65% of the sensory neurons that expressed PACAP following injury and i.p. injection of control IgG (data not shown). Once again no discernible difference in expression was found between animals receiving injury alone or injury and control IgG injection. Since the systemic injections of anti-BDNF after nerve transection did not have any obvious effect on PACAP mRNA expression, further quantifications of these tissues were not performed.

## Discussion

We and others have shown that the neuropeptide PACAP is markedly upregulated in sensory and motor neurons, in response to nerve transection, peripheral inflammation and compression injury [13], [15], [19], [21], [22], [25], [51], where it is believed to subserve roles in modulation of nociception [1]–[6], survival and regeneration of injured neurons [7]–[11]. The upregulation of PACAP expression in axotomized sensory and motor neurons parallels that of BDNF and both molecules can be readily modulated in a similar fashion by neurotrophin availability in injured sensory neurons ([15], [16], [19], [21], [26]). Thus, we investigated whether changes in endogenous BDNF are implicated in the regulation of PACAP expression in injured sensory and motor neurons. We reveal the novel finding that endogenous BDNF plays a critical role in induction of PACAP expression in sensory and motor neurons, but does not appear to be required to maintain PACAP expression once it has been induced in the one week injured neuron.

BDNF is normally expressed in small DRG sensory neurons that express the nerve growth factor receptor trkA [44], [52], [53]. In response to axotomy, expression changes rapidly and transiently and BDNF is expressed by a majority (∼80%) of the injured sensory neurons [26]. However, by one week after injury a phenotypic shift occurs whereby BDNF expression is decreased in smaller sized neurons, but increased in medium-large sized DRG neurons expressing trkB and trkC neurotrophin receptors [26], [27], [54]. BDNF expression in spinal cord motor neurons is also plastic, changing in response to injury e.g. ventral root avulsion, femoral nerve injury and sciatic nerve transection [29]–[31]. This rapid and flexible pattern of BDNF expression, and the fact that BDNF is the sole neurotrophin upregulated in sensory and motor neurons after peripheral nerve injury suggests a role for BDNF in modulation of the injury response. In rat retinal and cortical neurons, BDNF has been shown to increase levels of vasoactive intestinal polypeptide (VIP; another member of the VIP/secretin/glucagon/growth hormone-releasing factor superfamily to which PACAP also belongs) [55], [56]. In addition, BDNF expression patterns in subpopulations of DRG neurons and in spinal cord motor neurons resemble those of PACAP in response to injury and inflammation [19], [29]–[31], [57], [58], suggesting a possible causal effect with the altered PACAP expression.

We found PACAP to be expressed in roughly one fifth of intact DRG neurons, predominantly small sized neurons, consistent with previous findings [15], [16], [21], [22], [25]. In the present study we observed that 3 days after nerve transection there was a marked increase in PACAP mRNA expression, whereby PACAP mRNA was detected in ∼60% of the DRG neurons. This injury-induced increase was observed in all sizes of injured DRG neurons, and the expression pattern was similar with or without control IgG treatment, suggesting that mere IgG infusion does not alter the expression. However when anti-BDNF was infused for the 3 day injury period, the injury-induced increase in PACAP expression was markedly decreased with only ∼35% of the neurons now expressing detectable PACAP mRNA. The decreased incidence of expression was matched by a decrease in the level of PACAP mRNA expression/neuron. The reduction took place across all size ranges of DRG neurons, consistent with the size range of neurons expressing trkB, as well as neurons not expressing trkB [59]. This suggests that the effect of endogenous BDNF on the upregulation of PACAP mRNA expression in DRG neurons after axotomy is not restricted to neurons expressing the BDNF receptor, trkB. It might also be an indirect effect of BDNF signaling via truncated trkB receptors expressed on satellite cells [44], or via trkB expressing non-neuronal cells that in turn regulate the PACAP mRNA expression in non-trkB neurons through other mechanisms, e.g. upregulation of cytokines and growth factors. Alternatively, BDNF may initiate more global responses via the common neurotrophin receptor, p75, which is expressed by ∼80% of the DRG neurons and perineuronal cells as well as by other cell types [59], [60], and has been shown to influence signaling in response to BDNF [61]–[63]. Further, the inability of anti-BDNF treatment to reduce the level and incidence of expression to pre-injury levels suggests that either the affect of the antibody was not complete and/or BDNF is not the only injury-induced factor regulating injury-associated PACAP expression.

An alternate approach of interfering with BDNF signaling, by siBDNF injection, led to a similar, though not as robust, reduction of injury-induced PACAP expression. This further supports a role for endogenous BDNF in modulation of PACAP expression. Two major differences between this form of BDNF interference and anti-BDNF infusion may factor into this less robust response. First, the BDNF siRNA is likely only interfering with neuronal sources of BDNF since the fluorescently tagged siRNAs were only detected in neurons and not perineuronal or other glial cells (Fig. 8 in [35]). The anti-BDNF treatment on the other hand penetrated the entire DRG parenchyma and thus would have the potential to antagonize BDNF released by both neuronal and non-neuronal perineuronal sources. Secondly, the BDNF siRNA only targets BDNF mRNA, leaving existing stores of BDNF protein unaffected and available for release upon injury.

Delayed infusion of anti-BDNF, for 3 days one week after injury, did not affect the injury-induced increase in PACAP mRNA expression (∼66% of the DRG neurons expressed PACAP mRNA after control IgG infusions as compared to ∼67% after anti-BDNF infusions). This implies that BDNF is not necessary for maintaining an increased PACAP expression once it is induced.

We have previously examined the possibility that immunoneutralization of BDNF, by BDNF antagonizing antibodies, might lead to a non-selective effect on gene expression, downregulating expression of all genes. This theory was negated by the findings that infusion of anti-BDNF after injury had no obvious effect on the injury-induced expression of neuropeptide Y [35], a neuropeptide which has been previously shown not to be regulated by BDNF [64].

We have also recently found that anti-BDNF treatment impairs the intrinsic ability of 3 day injured sensory neurons to extend neurites when assayed in vitro following the in vivo manipulations [35]. DRGs from intact and injured rats treated with anti-BDNF or control IgG were dissociated and cultured for 24 hours after which mean neurite length was analysed and found to be markedly reduced in anti-BDNF treated animals. The effect was significant in injured neurons, suggesting that BDNF is critical for the enhanced intrinsic ability of injured sensory neurons to extend neurites. Having established in the present study that BDNF plays a critical role in induction of PACAP expression in DRG neurons after injury, supports that BDNFs axiogenic effect might be mediated at least in part via its effect on PACAP expression. PACAP has been shown to have neurite-stimulating abilities in different culture systems [10], [11], as well as mediating growth cone attraction on immature cells [65]. Axonal regeneration is initially impaired in motor neurons after injury in PACAP deficient mice, possibly due to changes in immunological responses such as inability to keep pro-inflammatory cytokine production/release in check, as well as a reduced production of anti-inflammatory cytokines [5], [7], further implicating PACAP in nerve regeneration. In addition, PACAP has also been found to modulate BDNF expression [9], and by preventing loss of BDNF expression in cultured rat cortical neurons PACAP can protect them from cell death induced by NMDA treatment (excitotoxin) or serum deprivation [66]. Whether a similar feedback loop exists in vivo in sensory and motor neurons remains to be determined.

In spinal cord motor neurons the impact of interfering with the BDNF axis was similar to what was found for DRG neurons. The incidence of injury-induced PACAP mRNA expression in the injured motor neurons (3 days after injury) was also reduced by immediate anti-BDNF treatment at the time of injury, decreasing from ∼55% to only 35% of the neurons expressing PACAP mRNA after anti-BDNF infusion. Delayed antibody infusion on the other hand had no discernible effect on PACAP mRNA expression in the spinal cord. Hence, BDNF seems to be important for the induction but not the maintenance of PACAP mRNA expression after nerve injury in motor neurons, as well as in sensory neurons. Even though the infused antibody was not readily detectable at the level of the lumbar enlargement, the route of how anti-BDNF is able to affect and mitigate PACAP expression in spinal cord ventral horn neurons after intrathecal infusion may be a result of retrograde axonal transport of small amounts of the infused antibody via the ventral root to the motor neurons. Specific receptor mediated retrograde transport of antibodies has been shown in motor neurons [67]. Another possibility is diffusion of the antibody into the spinal cord at the level of the lumbar enlargement, at levels not detectable by the immunohistochemical technique employed.

The lack of a measureable effect of i.p. anti-BDNF injections on PACAP mRNA expression in injured sensory neurons might be due to the systemic dose not being sufficient to neutralize the endogenous BDNF implicated in this response [37]. We suggest that the primary endogenous source of BDNF in regulation of PACAP mRNA expression in the DRG after nerve injury, is likely from the DRG neurons themselves where an upregulation in BDNF mRNA expression in response to injury mirrors that of PACAP [27], [54], [68]. This would be consistent with the reduction in PACAP expression that we observe in DRG neurons using immunoneutralization or siRNA infusion approaches.

In conclusion, the findings in this study indicate that the endogenous injury-associated BDNF upregulation is involved in the positive regulation of PACAP expression in sensory and motor neurons in response to the pathology. However, while BDNF is critical for the induction, it is not essential for the maintenance of injury-induced PACAP expression in these neurons. The relevance of the injury-associated upregulation in PACAP expression while not being fully elucidated at this point, provides a novel target in manipulation of regenerative states.
